# Echocardiographic Evaluation of the Thoracic Aorta: Tips and Pitfalls

**DOI:** 10.1055/s-0041-1724005

**Published:** 2021-10-04

**Authors:** Krishna Upadhyaya, Ifeoma Ugonabo, Keyuree Satam, Sarah C. Hull

**Affiliations:** 1 Section of Cardiovascular Medicine, Columbia St. Mary's Hospital, Milwaukee, Wisconsin; 2 Division of Cardiovascular Diseases, University of Tennessee-Methodist, Knoxville, Tennessee; 3 Section of Cardiovascular Medicine, Yale University School of Medicine, New Haven, Connecticut

**Keywords:** echocardiography, aorta, dissection, bicuspid aortic valve

## Abstract

By convention, the ascending aorta is measured by echo from leading edge to leading edge. “Leading edge” connotes the edge of the aortic wall that is closest to the probe (at the top of the inverted “V” of the ultrasound image). By transthoracic echo (TTE), the leading edges are the outer anterior wall and inner posterior wall. By transesophageal echo (TEE), the leading edges are the outer posterior wall and inner anterior wall. Aortic measurements should be taken (by convention) in diastole (when the aorta is moving least). Simple TTE is 70 to 85% sensitive in diagnosing ascending aortic dissection. TEE sensitivity approaches 100%, though the tracheal carina imposes a blind spot on TEE, impeding visualization of distal ascending aorta and proximal aortic arch. While computed tomography angiography may be superior for defining full anatomic extent of aortic dissection, echocardiography is superior in assessing functional consequences such as mechanism and severity of aortic regurgitation, evidence of myocardial ischemia when complicated by coronary dissection, or evidence of tamponade physiology when pericardial effusion is present. Reverberation artifact can mimic a dissection flap. A true flap moves independently of the outer aortic wall which can be confirmed by M-mode. Color flow respects a true flap but does not respect a reverberation artifact. Assessment for bicuspid aortic valve (BAV) morphology should be done in systole, not diastole. In diastole, when the valve is closed, the raphé can make a bicuspid valve appear trileaflet. Doming in the parasternal long axis (PLAX) view and an eccentric closure line on PLAX M-mode should also raise suspicion for BAV.

## Introduction

Aortic disease can present as a multitude of conditions, ranging from asymptomatic aneurysms to life-threatening acute aortic syndromes. While a significant cause of morbidity and mortality, the incidence of aortic disease is likely underestimated due to some processes being clinically silent. Thoracic aortic aneurysms (TAA) are estimated to have an annual incidence of 5 to 10 cases/100,000 patient years, with more acute symptomatic syndromes like thoracic aortic dissection having an incidence of 3 to 4 cases/100,000 patient years. 1 2 In acute aortic syndromes, and especially in asymptomatic diseases where there may not be any physical examination cues, echocardiography plays an important role in diagnosis and serial evaluation. Standard transthoracic echocardiography (TTE) obtains certain views of the aorta and is useful not only in diagnosis but also in follow-up screening. Transesophageal echo (TEE) also plays a vital role in obtaining a more comprehensive view of the aorta, especially when transthoracic views are limited. In this review, we will highlight echocardiographic evaluation of the normal aorta and selected aortic pathologies, including tips to improve imaging quality and pitfalls to avoid that may impede accurate assessment.

## Echocardiographic Evaluation of the Normal Aorta

The aorta is divided into five anatomic segments: (1) aortic root, (2) tubular ascending aorta, (3) aortic arch, (4) descending aorta, and (5) abdominal aorta. The aortic root is further subdivided into the sinuses of Valsalva and the sinotubular junction, which is the intersection between the root and the tubular ascending aorta. The aortic annulus is the most proximal portion of the aortic root, where the leaflets of the aortic valve are inserted. Just distal to that are the sinuses of Valsalva from which the coronary arteries originate. The aorta as a whole serves as a dynamic elastic conduit connecting the cardiac pump to the rest of the organs in the body and thereby delivers oxygenated blood. Evaluation of the aorta is part of the standardized protocol when performing a TTE, though it is important to note that not every aforementioned segment may be adequately visualized with this modality.

**Fig. 1:**
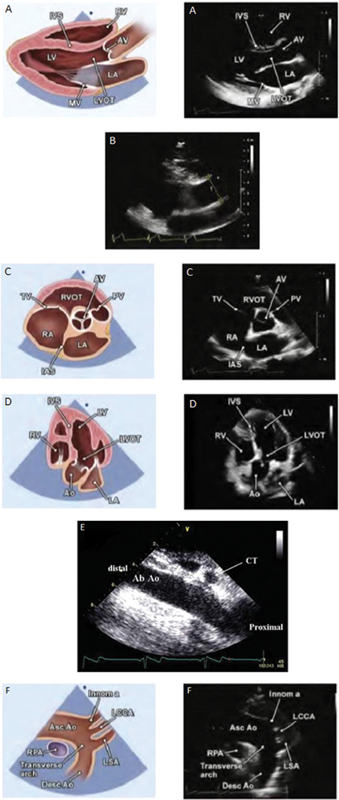
Canonical views on transthoracic echo (TTE): ( **A** ) parasternal long axis with anatomic and TTE images, ( **B** ) parasternal long axis TTE image up a rib space showing ascending aorta, ( **C** ) parasternal short axis with anatomic and TTE images, ( **D** ) apical five chambers with anatomic and TTE images, ( **E** ) subcostal TTE image, ( **F** ) suprasternal with anatomic and TTE images. Images ( **A–D** ) and ( **F** ) adapted from Mitchell et al 3 and image ( **E** ) from Evangelista et al. 13 Ab Ao, abdominal aorta; Asc Ao, ascending aorta; AV, aortic valve; CT, computed tomography; Desc Ao, descending aorta; IAS, interartrial septum; Innom a, innominate artery; IVS, interventricular septom; LA, left atrium; LAX, long axis; LCCA, left common carotid artery; LSA, left subclavian artery; LV, left ventricle; LVOT, left ventricular outflow tract; ME, mid esophageal; MV, mitral valve; PV, pulmonic valve; RA, right atrium; RV, right ventricle; RVOT, right ventricular outflow tract; SAX, short axis; TV, tricuspid valve; UE, upper esophageal.

The canonical views in which the aorta is visualized with TTE are the parasternal long axis (PLAX), parasternal short axis, apical view, subcostal view, and suprasternal view, typically in that order. 3 The PLAX is viewed by placing the patient in the left lateral decubitus position and the ultrasound probe at the third or fourth intercostal space, adjacent to the sternum on the left side, with the transducer marker pointing to the right shoulder. In this window, the parts of the aorta visualized in longitudinal cross-section are the aortic annulus, sinuses of Valsalva, sinotubular junction, and proximal part of the tubular ascending aorta ( Fig. 1A ). The descending aorta can also be visualized just below the left atrium, though in transverse cross-section. A tip to better visualize the tubular ascending aorta is remaining adjacent to the sternum but going up a rib space. This window allows for better visualization and is used for measurement of the proximal ascending aorta ( Fig. 1B ). If the probe is rotated 90 degrees with the marker facing the left shoulder, the left ventricle appears in transverse cross section (short axis), and if the probe tail is tipped down, the aortic annulus appears in transverse cross-section with visualization of the aortic valve ( Fig. 1C ). This is useful in helping evaluate the number of aortic valve leaflets. In the apical five and three chamber view, the aortic annulus and sinuses of Valsalva can be visualized ( Fig. 1D ), though size measurements are not typically made in this view as the PLAX is preferred given closer proximity to the probe. This window is useful in obtaining functional information about the valve, such as stenosis or regurgitation, given better alignment with the spectral Doppler interrogation. The subcostal view is helpful in evaluating the descending aorta, which can be visualized by tipping the probe tail up after seeing the inferior vena cava with the marker pointing in the 3 o'clock position ( Fig. 1E ). Finally, in the suprasternal view, the aortic arch can be visualized, including the three main branches of the arch if image quality is excellent ( Fig. 1F ).

**Fig. 2:**
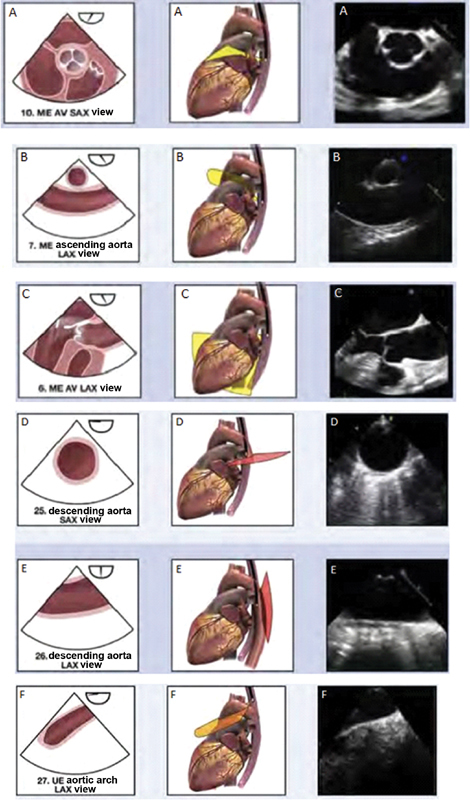
Canonical views on transesophageal echo (TEE): ( **A** ) mid-esophageal view at 45 degrees (short axis) with anatomic, three-dimensional (3D) and TEE images, ( **B** ) mid-esophageal view at 100 degrees of the ascending aorta with anatomic, 3D and TEE images ( **C** ) mid-esophageal view at 135 degrees (long axis) with anatomic, 3D and TEE images, ( **D** ) mid-esophageal view at 0 degrees (short axis) of the descending aorta with anatomic, 3D and TEE images, ( **E** ) mid-esophageal view at 90 degrees (long axis) of the descending aorta with anatomic, 3D and TEE images, ( **F** ) upper esophageal view at 0 degrees of the aortic arch with anatomic, 3D and TEE images. AV, aortic valve; LAX, long axis, ME, mid esophageal; SAX, short axis; UE, upper esophageal. Image courtesy: Hahn et al. 4

The canonical views in which the aorta is visualized on TEE include mid-esophageal and upper-esophageal views at different plane angles. 4 At approximately 45 degrees in the mid-esophageal view, the aortic valve can be seen in transverse cross-section, which is helpful for direct visualization to determine the number of leaflets ( Fig. 2A ). If the probe is pulled out a few centimeters, at approximately 100 degrees and with some anteflexion, the proximal ascending aorta can be laid out in a longitudinal view for diameter measurement ( Fig. 2B ). In the mid-esophageal view at 135 degrees, the aortic valve can be visualized in long axis, which can be used to measure the left ventricular outflow tract, sinuses of Valsalva, and sinotubular junction, as well as to assess aortic valve function ( Fig. 2C ). Finally, in the mid-esophageal view, the probe is rotated in clockwise or counterclockwise fashion until the descending aorta can be viewed, in 0 degrees as short axis and 90 degrees as long axis ( Fig. 2D, E ). As the probe is pulled out, in the upper esophagus, the aortic arch can be visualized as well ( Fig. 2F ).

**Fig. 3:**
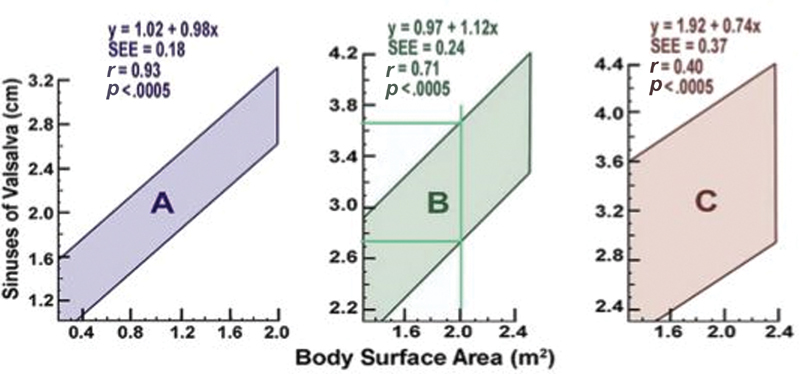
Normal ranges of Sinuses of Valsalva diameter with respect to body surface area across three age groups (left to right: 1–15 years, 20–39 years, and >40 years). SEE, standard error of the estimate. Image courtesies: Roman et al 5 and Goldstein et al. 8

When measuring the aorta by echocardiography, it is important to emphasize that normal aortic size can vary from person to person based on certain patient characteristics. Roman et al 5 studied the aortic root diameters of 135 normal adults and 52 normal infants and children and compared several characteristics including age, gender, body habitus, blood pressure, and stroke volume. Of these characteristics, age and body surface area (BSA) were found to be the most significant factors in determining normal aortic size. Fig. 3 shows normal ranges of the sinuses of Valsalva diameter for three different age groups: Fig. 3A is for ages 1 to 15 years, Fig. 3B is for 20 to 39 years, and Fig. 3C is for ≥40 years. Based on this chart, for example, a 50-year-old patient with a BSA of 2.2 would have an upper limit of normal of 4.2 cm, whereas a 30-year-old patient with a BSA of 1.6 would have an upper limit of normal of 3.2 cm.

**Fig. 4:**
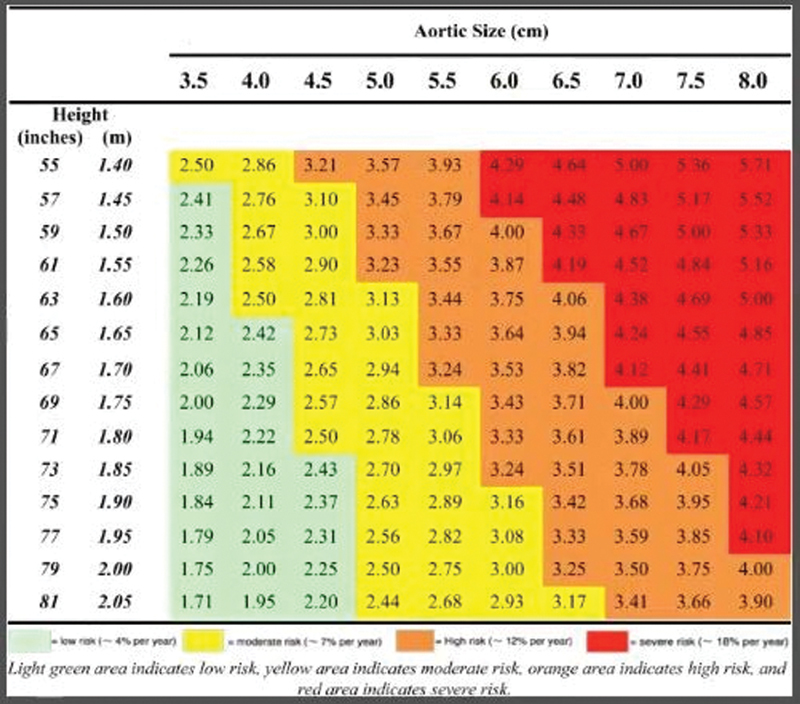
Risk of complications (aortic dissection, rupture, and death) in patients with ascending aortic aneurysm as a function of aortic diameter (horizontal axis) and height (vertical axis), with the aortic height index given within the figure. Image courtesy: Zafar et al. 7

Normal values of the ascending aorta are also based on certain patient characteristics. Davies et al 6 followed 803 patients with thoracic aortic aneurysms with serial multimodal imaging and found that aortic diameter indexed to BSA was a more important predictor of outcomes than absolute diameter. In a more recent study, the same group indexed aortic size to height in 780 patients and found that height-based ratio was as good or better at predicting outcomes ( Fig. 4 ). 7 Therefore, it is important not to fall into the pitfall of “one size fits all” for normal aortic size dimensions.

**Fig. 5:**
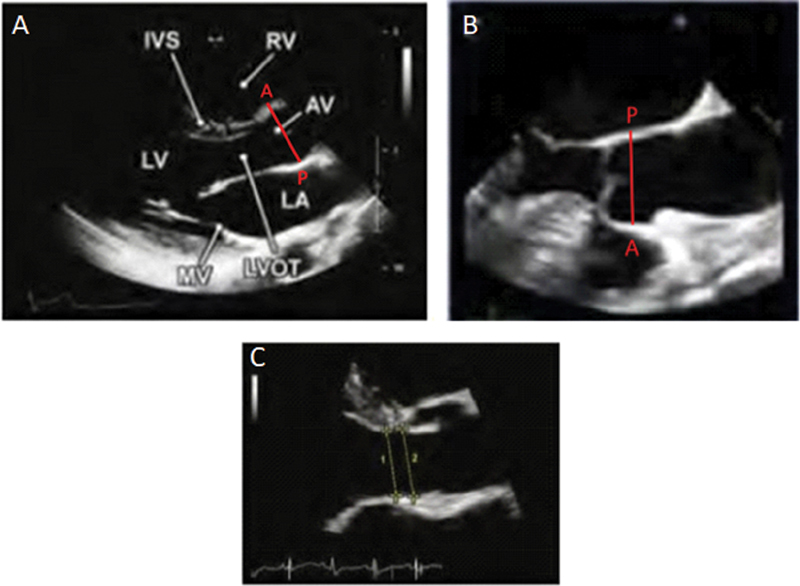
( **A** ) Parasternal long axis on transthoracic echo (TTE) showing leading edge to leading edge measurement, from outside the anterior wall to the inside of the posterior wall, ( **B** ) transesophageal echo (TEE) mid-esophageal view at 135 degrees (long axis) showing leading edge to leading edge measurement, from outside the posterior wall to the inside of the anterior wall, ( **C** ) parasternal long axis on TTE showing inner edge to inner edge measurement of the (1) LVOT and (2) aortic annulus in midsystole with open leaflets. A, anterior, AV, aortic valve; IVS, interventricular septum; LA, left atrium; LV, left ventricle; LVOT, left ventricular outflow tract; MV, mitral valve; P, posterior; RV, right ventricle. Red lines added for this figure to demonstrate proper measuring technique. Image courtesy: Mitchell et al. 3

It is also important to emphasize the proper technique for measuring the normal size of the aorta. There are three key techniques that should be followed to ensure accurate measurement: (1) timing of the cardiac cycle, (2) edges of measurement, and (3) echocardiographic windows. 8 With respect to timing of the cardiac cycle, all measurements of the aorta except the aortic annulus are recommended to be done in end diastole. The aorta has elastic properties that enable it to accommodate the ejection of blood from the left ventricle, and thus varies slightly in diameter between systole and diastole. Therefore, consistent measures must be used to compare with normal values for each study, as well as to compare follow-up studies in a single patient. With respect to edges of measurements, aortic root and ascending aorta measurements should be done from leading edge to leading edge. For TTE, this means measurement of the outside of the anterior wall to the inside of the posterior wall, and for TEE, this would be reversed since the probe is behind the heart, that is, outside of the posterior wall to the inside of the anterior wall ( Fig. 5A, B ). Leading edge to leading edge was originally recommended by the American Society of Echocardiography (ASE) in 1978 due to the belief that this technique reduces inaccuracies in measurement that could arise from blooming artifact due to the aortic wall being a bright reflector. 9 Large studies that were done after those ASE recommendations and multiple guidelines have since used the leading edge to leading edge in end-diastole technique, and thus most available prognostic data have stemmed from this convention. Moreover, Muraru et al 10 performed aortic measurements using inner edge to inner edge verses leading edge to leading edge with two-dimensional (2D) echocardiography in 218 healthy volunteers and found that inner edge measurements produced significantly smaller diameters as compared with leading edge diameters. 10 The only exception to this convention, however, is the aortic annulus, which is measured from inner edge to inner edge, where the leaflets insert, in midsystole when the leaflet tips are open ( Fig. 5C ). Finally, the TTE window that measurements are done is the PLAX, where the aorta lies perpendicular to the ultrasound probe.

Echocardiography is a useful imaging modality because it is cost effective, portable, and widely available. Other than echocardiography, however, computed tomography (CT) and magnetic resonance imaging (MRI) can also be used as multimodal approaches to imaging the aorta, though these techniques may not be as cost effective or accessible. CT angiography (CTA) can provide a rapid, noninvasive tool that allows visualization of the entire aorta, though it exposes the patient to radiation and contrast and cannot provide functional information about the aortic valve. MRI can also provide visualization of the entire aorta without any radiation exposure, though it is costly and less widely available, and the patient has to remain still in a supine position for a much longer time. Interestingly, these imaging modalities use an inner edge to inner edge measuring technique. When comparing echocardiographic measurement convention, there is good correlation with inner edge to inner edge measurements in CT/MRI. In fact, the three modalities were compared by Rodríguez-Palomares et al 11 in 2016 in a study of 140 patients with aortic disease. They found that when comparing leading edge to leading edge technique in TTE to inner edge to inner edge technique in CT/MRI, there was good accuracy and reproducibility of aortic diameter estimation. When inner edge to inner edge was used in TTE, however, it underestimated the diameters as compared with CT/MRI. It is therefore worth highlighting that in addition to TTE, CT and MRI can be useful tools for physicians to utilize in aortic evaluation, especially when visualization of the entire aorta is necessary as this cannot be achieved with TTE alone.

## Echocardiographic Evaluation of Selected Aortic Pathologies

### Aortic Dissection



**Video 1** ( **A** ) Transesophageal echo (TEE) view of the aorta in transverse cross section showing a dissection flap with a true lumen and a false lumen that is thrombosed, ( **B** ) TEE view from Video 1A with color Doppler, showing respect of the flap boundaries and no flow in the thrombosed false lumen, ( **C** ) TEE view of the aorta in transverse cross section showing a reverberation artifact “flap,” ( **D** ) TEE view from Video 1C with color Doppler, showing color traversing the entirety of the lumen. Videos from the Yale Echocardiography Laboratory. 2D, two-dimensional; bpm, beats per minute.


Aortic dissection, which falls under the category of acute aortic syndromes, occurs when there is a tear in the intimal layer of the aorta, and pulsatile blood flow enters causing a separation of the layers. Subsequently, the tear can propagate, resulting in two lumens of blood flow, a true and false lumen. Aortic dissections can be categorized based on the Stanford classification system into Type A and Type B, with Type A defined as dissections of the ascending aorta ± the arch and descending aorta, and Type B defined as dissection of the aorta below the level of the left subclavian artery only. 12 Type A dissections are particularly dangerous, as they can propagate into the coronary arteries causing myocardial infarction, disrupt the aortic valve resulting in significant valvular regurgitation, or extend into the pericardium resulting in acute pericardial effusion and cardiac tamponade. While CT has traditionally been the initial diagnostic test of choice, echocardiography provides valuable information in this condition, and the two modalities can be used in combination for evaluation and treatment planning. CT has the advantage that it can allow visualization of the entire aorta and branch vessels, but it does not provide any functional data that echocardiography can provide such as the mechanism and severity of aortic regurgitation, presence of wall motion abnormalities to suggest coronary involvement, or evidence of hemodynamically significant pericardial effusion. Furthermore, in patients who are hemodynamically too unstable to go to a CT scanner, or cannot get contrast due to renal disease, TEE provides a portable diagnostic tool that can be performed at the bedside with a sensitivity of 86 to 100% and a specificity of 90 to 100%. 13 It is important to keep in mind, however, that TEE has a blind spot at the distal ascending aorta and proximal aortic arch because the air-filled tracheal carina interferes with ultrasound beam propagation from the esophagus to the heart. Table 1 describes the different criteria for diagnosis of dissection that can be detected by echocardiography.

**Table 1:** Role of echocardiography in detecting evidence of aortic dissection and echocardiographic definitions of main findings (adapted from Goldstein et al 8 )

Diagnostic goals	Definition by echocardiography
Identify presence of a dissection flap	Flap diving two lumens
Define extension of aortic dissection	Extension of the flap and true/false lumens in the aortic root (ascending/arch/descending abdominal aorta)
Identify true lumen	Systolic expansion, diastolic collapse, systolic jet directed away from the lumen, absence of spontaneous contrast, and forward systolic flow
Identify false lumen	Diastolic diameter increase, spontaneous contrast and or thrombus formation, and reverse/delayed or absent flow
Identify presence of false luminal thrombosis	Mass separated from the intimal flap and aortic wall inside the false lumen
Localize entry tear	Disruption of the flap continuity with fluttering or ruptured intimal borders; color Doppler shows flow through the tear
Assess presence, severity and mechanisms of aortic regurgitation	Anatomic definition of the valve (bicuspid, degenerated, and normal with/without prolapse of one cusp); dilation of different segments of the aorta; flap invagination into the valve; and severity by classic echocardiographic criteria
Assess coronary artery involvement	Flap invaginated into the coronary ostium, flap obstructing the ostium, absence of coronary flow, and new regional wall motion abnormalities
Assess side-branch involvement	Flap invaginated into the aortic branches
Detect pericardial and/or pleural effusion	Echo-free space in the pericardium/pleura
Detect signs of cardiac tamponade	Classic echocardiographic and Doppler signs of tamponade

**Fig. 6:**
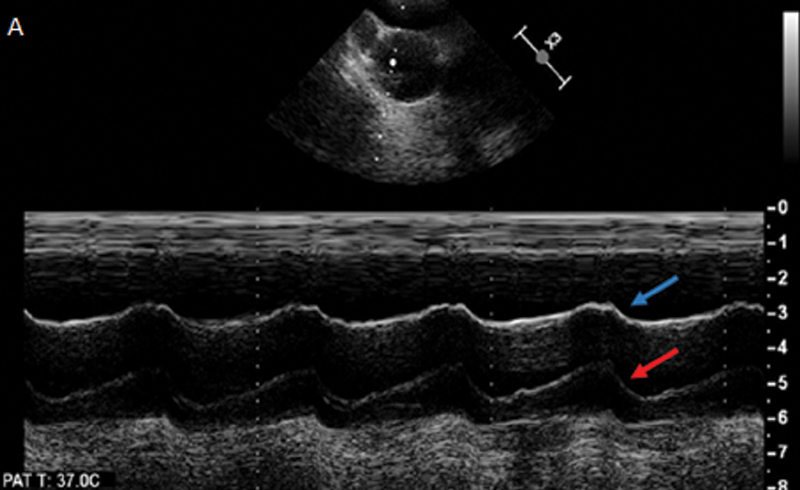
M-mode of the aorta in cross section on transesophageal echo showing reverberation artifact. The “dissection flap” (red arrow) shows perfect concordance with the aortic wall (blue arrow), suggesting artifact rather than true dissection. Image courtesy: Yale Echocardiography Laboratory.

One of the pitfalls of aortic imaging by echocardiography to note is reverberation artifact which can create the false appearance of a dissection flap. A reverberation artifact occurs when there is a strong reflector of ultrasound beams within the scanning sector, such as the wall of the aorta, which causes the ultrasound beams to bounce back and forth causing the initial structure to reappear with weaker intensity, farther away from the probe. 14 This can result in a diagnostic dilemma in which the reader may confuse an artifact for a dissection flap. There are several tips and tricks that can be used to distinguish artifact from true dissection. Color Doppler imaging can help with this distinction, as it will respect the boundaries of a true dissection flap and show two distinct areas of different color flow in the true lumen and the false lumen ( Video 1A, B ; available in the online version only). 15 If the false lumen is thrombosed, it may show minimal or no color flow. However, color Doppler flow patterns will traverse the entirety of the aortic lumen without respecting a “flap” that is really reverberation artifact ( Video 1C, D ; available in the online version only). M-mode interrogation can also be performed through the aorta to see if there is independent motion of the “flap.” A reverberation artifact will not demonstrate independent motion and will perfectly mimic movement of the aortic wall, whereas a true dissection flap should demonstrate independent fluttering motion as blood flows through it ( Fig. 6 ).

Lastly, contrast agents can be used to follow blood flow, and visualization of different lumens can be identified. Similar to color flow, contrast will either follow or not follow the boundaries of the flaps in the cases of true dissection versus artifact, respectively. While TTE has less sensitivity than TEE for dissection, the addition of contrast can increase TTE sensitivity from 70–85 to 93% assuming sufficient image quality. 8 13

### Bicuspid Aortic Valve



**Video 2** ( **A** ) Parasternal long axis transthoracic echo (TTE) view showing doming of the aortic valve leaflets, ( **B** ) parasternal long axis TTE view with color Doppler showing an eccentric jet of aortic regurgitation. Source Yale Echocardiography Laboratory.


**Fig. 7:**
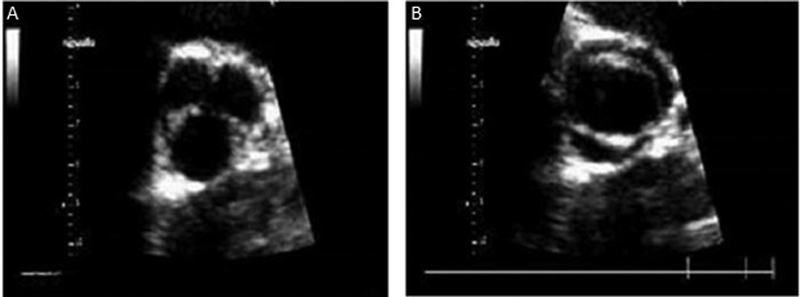
( **A** ) Aortic valve during diastole showing the appearance of three leaflets, ( **B** ) same aortic valve in systole showing true bicuspid valve. Image courtesy: Santarpia et al. 17

Bicuspid aortic valve (BAV) is a congenital cardiac abnormality in which there are two aortic valve cusps instead of three. It has a prevalence of 1 to 2%, with the most common type being fusion of the right and left coronary cusp, accounting for 70 to 80% of BAVs. 16 Diagnosis of this condition is of paramount importance because these valves can degenerate at a much younger age leading to symptomatic stenosis or regurgitation, and patients can have associated aortic aneurysms with literature reports of prevalence varying from 20 to 84%. 16 The primary mode of diagnosis of BAV is echocardiography. The best view to diagnose BAV is in the parasternal short axis. It is important to highlight that one pitfall is viewing the valve in diastole as opposed to systole. If it is visualized in diastole when the valve is closed, it can still appear trileaflet even if it is bicuspid due to the presence of a raphé or fusion line between two leaflets. Therefore, it should be viewed in systole when the valve is open, to visualize the commissural fusion with raphé and “fish mouth” or football-shaped appearance of the valve orifice ( Fig. 7 ). 17

**Fig. 8:**
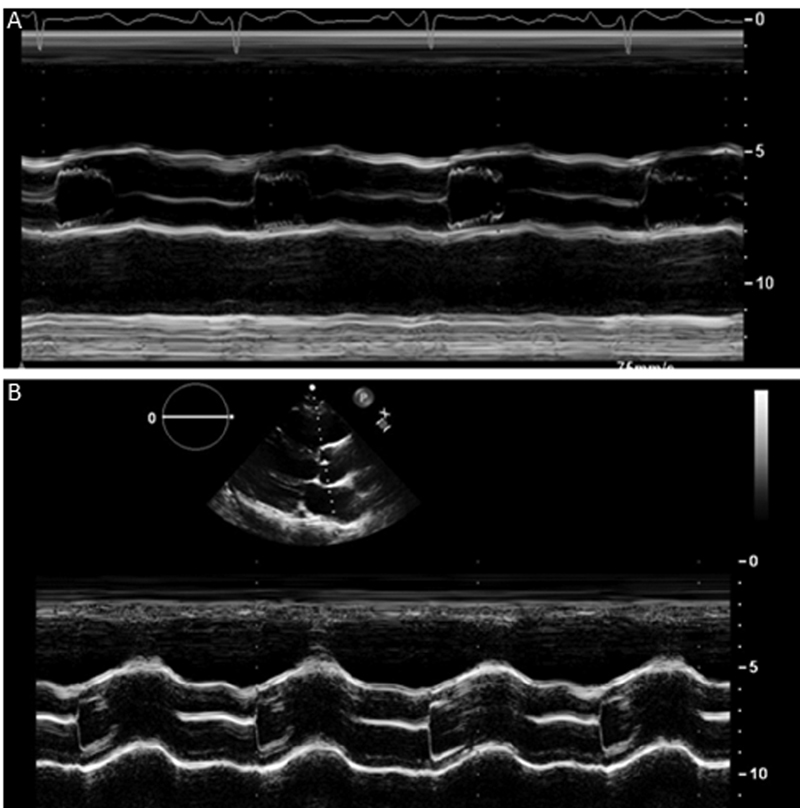
( **A** ) Central closure suggestive of a tricuspid aortic valve, ( **B** ) eccentric closure suggesting bicuspid aortic valve. Image courtesy: Yale Echocardiography Laboratory.

Another tip to help distinguish bicuspid from trileaflet valve is M-mode interrogation through the aortic valve in PLAX. Normally, the closure line of the aortic valve in diastole should be in the center of the aorta, but with bicuspid valves, one of two valve leaflets is usually bigger, and so the closure line will be eccentric ( Fig. 8A, B ). 18 Furthermore, in the PLAX, doming of the leaflets can be seen ( Video 2A ; available in the online version only). Finally, a tip off suggesting possible bicuspid valve is eccentric aortic regurgitation ( Video 2B ; available in the online version only).

## Conclusion

Echocardiography is a vital imaging modality in the evaluation of the thoracic aorta. It is important to keep in mind standard techniques for visualization and measurements, such as leading edge to leading edge, end diastole, and appropriate window, to accurately diagnose and follow-up patients. Different techniques, such as M-mode, color Doppler flow, or contrast echocardiography, can be useful tools to help distinguish between true disease and artifact. Clinicians should be aware of pitfalls in interpreting echocardiographic studies that may lead to misdiagnosis or delay in diagnosis. CT and MRI are other multimodal approaches for diagnosis and evaluation of aortic pathology when visualization on TTE or TEE is suboptimal or further imaging is required.
